# A Combination of Coffee Compounds Shows Insulin-Sensitizing and Hepatoprotective Effects in a Rat Model of Diet-Induced Metabolic Syndrome

**DOI:** 10.3390/nu10010006

**Published:** 2017-12-22

**Authors:** Pedram Shokouh, Per Bendix Jeppesen, Kjeld Hermansen, Natalja P. Nørskov, Christoffer Laustsen, Stephen Jacques Hamilton-Dutoit, Haiyun Qi, Hans Stødkilde-Jørgensen, Søren Gregersen

**Affiliations:** 1 Department of Endocrinology and Internal Medicine, Aarhus University Hospital, Tage-Hansens Gade 2, 8000 Aarhus C, Denmark; per.bendix.jeppesen@clin.au.dk (P.B.J.); kjeld.hermansen@aarhus.rm.dk (K.H.); soeren.gregersen@aarhus.rm.dk (S.G.); 2 The Danish Diabetes Academy, Odense University Hospital, Kløvervænget 10, 5000 Odense C, Denmark; 3 Department of Animal Science, Aarhus University, 8830 Tjele, Denmark; natalja.norskov@anis.au.dk; 4 MR Research Centre, Aarhus University Hospital Skejby, 8200 Aarhus N, Denmark; cl@clin.au.dk (C.L.); qi@clin.au.dk (H.Q.); hsj@clin.au.dk (H.S.-J.); 5 Institute of Pathology, Aarhus University Hospital Skejby, 8200 Aarhus N, Denmark; stephami@rm.dk

**Keywords:** coffee, dietary supplements, metabolic syndrome X, non-alcoholic fatty liver disease, adiponectin, hyperpolarized magnetic resonance spectroscopy

## Abstract

Since coffee may help to prevent the development of metabolic syndrome (MetS), we aimed to evaluate the short- and long-term effects of a coffee-based supplement on different features of diet-induced MetS. In this study, 24 Sprague Dawley rats were divided into control or nutraceuticals groups to receive a high-fat/high-fructose diet with or without a mixture of caffeic acid (30 mg/day), trigonelline (20 mg/day), and cafestol (1 mg/day) for 12 weeks. An additional 11 rats were assigned to an acute crossover study. In the chronic experiment, nutraceuticals did not alter body weight or glycemic control, but improved fed hyperinsulinemia (mean difference = 30.80 mU/L, *p* = 0.044) and homeostatic model assessment-insulin resistance (HOMA-IR) (mean difference = 15.29, *p* = 0.033), and plasma adiponectin levels (mean difference = −0.99 µg/mL, *p* = 0.048). The impact of nutraceuticals on post-prandial glycemia tended to be more pronounced after acute administration than at the end of the chronic study. Circulating (mean difference = 4.75 U/L, *p* = 0.014) and intrahepatocellular alanine transaminase activity was assessed by hyperpolarized-^13^C nuclear magnetic resonance NMR spectroscopy and found to be reduced by coffee nutraceuticals at endpoint. There was also a tendency towards lower liver triglyceride content and histological steatosis score in the intervention group. In conclusion, a mixture of coffee nutraceuticals improved insulin sensitivity and exhibited hepatoprotective effects in a rat model of MetS. Higher dosages with or without caffeine deserve to be studied in the future.

## 1. Introduction

According to the WHO’s Global Health Observatory [1], at least 2.8 million people die annually worldwide from the complications of excessive body fat. Calorie intake beyond the buffering capacity limit of adipose tissues will shift the lipid influx to the non-adipose tissue (ectopic) depots [2]. The ectopic deposition of triglycerides triggers a series of cardiometabolic perturbations, which are grouped into a diagnostic entity known as metabolic syndrome (MetS) [3]. However, obesity is a modifiable cardiometabolic risk factor that can be managed effectively by sustainable changes in lifestyle and diet [4]. As part of a healthy diet, coffee may exert protective effects toward various health outcomes [5]. As observational data suggest, coffee intake is inversely associated with all-cause and cardiovascular mortality [6]. Furthermore, coffee has shown potential in enhancing weight loss [7] and protecting against the development of the MetS and its hepatic component: nonalcoholic fatty liver disease (NAFLD) [8]. Yet, the range of compounds responsible for those effects has remained elusive. 

With moderate consumption (4–6 cups/day), some, but not all, coffee constituents reach bioactive levels in the human body. Among them are chlorogenic acids, caffeine, trigonelline, and diterpenes [7]. Since decaffeinated coffee largely retains its metabolic efficacy [9,10], it is hypothesized that caffeine may not be essential for most of the positive cardiometabolic effects attributed to long-term coffee consumption. Esters of caffeic acid, e.g., caffeoylquinic acids (CQAs), are widely studied, and have demonstrated potent antioxidant and anti-adipogenic effects. These compounds can delay glucose absorption, alleviate insulin resistance, and inhibit hepatic triglyceride accumulation [11]. It was proposed that the caffeic acid moiety of CQAs is mainly responsible for the bioactivity of the molecule [12]. It also exhibits a much higher bioavailability compared to CGAs [13]. Consequently, we chose to include caffeic acid instead of CQA isomers (e.g., 5-CQA) in our study, together with trigonelline and cafestol, aiming to compose a chemical combination with potential metabolic effects similar to decaffeinated coffee. Recently, we demonstrated that cafestol (a diterpene present in unfiltered coffee) has insulinotropic effects on pancreatic beta-cells, stimulates glucose uptake in human skeletal muscle cells [14], and improves glycemic control in vivo [15]. 

We hypothesized that coffee’s wide range of effects on different aspects of glucose and lipid metabolism cannot be attributed to a single compound, but rather to a combination of key constituents. To trial this hypothesis, we combined chemicals from three main groups of coffee compounds and studied their effects on weight gain, insulin resistance (IR), and liver steatosis in a rat model of diet-induced MetS.

## 2. Materials and Methods 

### 2.1. Animal Model

Sprague Dawley rats (*n* = 35) were purchased from Taconic A/S (Ejby, Denmark), caged individually, and allowed to acclimatize for a week with free access to regular food and water. Animals were kept in a facility with reversed 12-h light-dark cycles between 21 °C and 24 °C, and relative humidity of 55–70%. Animals were maintained, handled, and terminated by qualified personnel in keeping with the EU provisions and the Danish Act on the Protection of Animals. Regular inspections were performed by the Danish Animal Experiments Inspectorate (registration number: 2015-15-0201-00592). 

### 2.2. Diet

The diet consisted of a custom-made version of D12492 (D16030909, Research Diets, Inc., New Brunswick, NJ, USA), in which the whole carbohydrate content was replaced by fructose, and was used as a high-fat/high-fructose (HFHF) diet to induce obesity and IR. This diet contained 35% *w*/*w* fat (60% of total calories), 26% *w*/*w* protein, and 26% *w*/*w* fructose, and was dispensed every other day to minimize rancidity. High fructose in HF food increases its similarity to typical Western diet, and supposedly potentiates its metabolically deleterious effects [16].

### 2.3. Intervention

The intervention comprised of dietary supplementation of a fixed dosage of 30 mg (71.4 mg/kg) of caffeic acid, 20 mg (47.6 mg/kg) of trigonelline, and 1 mg (2.4 mg/kg) of cafestol per day per rat. Caffeic acid and trigonelline were dissolved in a fixed amount of 80 °C water (40–50 mL/rat, depending on the consumption) and given to the rats as drinking water after cooling down. Chemical solutions were made every day to avoid significant chemical degradation. Cafestol is not soluble in water and was hence dispensed once daily via a small food bolus prepared by dissolving 1 mg of cafestol in 50 µL of absolute ethanol and adding it to a small piece of puffed corn. The intervention started simultaneously with HFHF dieting. An interspecies dose conversion based on a body surface area (BSA) normalization method was performed, assuming the approximate BSA of a rat to be 0.06 m^2^ and of human to be 1.69 m^2^. Caffeic acid, trigonelline hydrochloride, and cafestol acetate were purchased from Sigma-Aldrich (St. Louis, MO, USA).

### 2.4. Chronic Study Design

For this study, 24 six-week-old rats were randomly assigned into two groups: control, and intervention (referred to as the nutraceuticals group hereafter). During the 12-week intervention period, body weight and food consumption were measured every second week, and blood glucose was monitored at weeks 5, 9, and 12 using a glucometer (FreeStyle Precision, Abbott GmbH & Co. KG, Wiesbaden, Germany). A blood sample was obtained at baseline (tail), week 6 (tail), and endpoint (retro-orbital). To convert whole blood to plasma values, glucometer-measured glucose was multiplied by 1.12. The homeostatic model assessment-insulin resistance (HOMA-IR) at week 6 was calculated by the formula: glucose mmol/L × insulin mU/L/22.5 [17]. At week 10, hyperpolarized nuclear magnetic resonance (NMR) imaging was performed (described below). After one week of recovery, rats underwent an oral glucose tolerance test (OGTT) by gastric gavage of 2 g/kg of d-glucose. Blood glucose was measured before, at 30, 60, 120, and 180 min after the gavage. At the end of the study, rats were anesthetized by intraperitoneal injection of 50 mg/kg of sodium pentobarbital. Retro-orbital blood samples were obtained and urine was collected from the bladder. Liver and pancreas tissue was harvested and fixed by immersion in 4% formaldehyde and stored at 4 °C until paraffin embedding (see the histopathological examination section). Rats were terminated by exsanguination at the end of the procedure.

### 2.5. Acute Study Design

In the study, 11 rats (average age = 12 weeks) were maintained under the same conditions and diet as the rats from our chronic study for 12 weeks. After 8 h of fasting, animals were randomly divided into two groups, and received pellets (either plain or containing half of the daily dosage of coffee nutraceuticals) in the chronic study 90 min before an OGTT (the protocol is described above). After a wash-out period of one week, the groups were switched and the same protocol was followed in a cross-over fashion. At the end of the study, rats were euthanized by intraperitoneal injection of sodium pentobarbital (100 mg/kg).

### 2.6. Immunoassays and Reagents

Whole blood glycated hemoglobin (HbA_1C_) (collected in EDTA tubes), and plasma glucose, lipoproteins, and alanine aminotransferase (ALT) were quantified by Cobas c111 analyzer (Roche Diagnostics, Mannheim, Germany). A sensitive rat RIA kit (EMD Millipore, Billerica, MA, USA) was used to measure insulin, and a NEFA-HR2 assay (Wako Chemicals GmbH, Neuss, Germany) was performed to determine non-esterified fatty acids (NEFAs) concentrations. Plasma adiponectin was quantified by AssayMax ELISA kit (Assaypro, St. Charles, MO, USA) and IL1b by Cloud-Clone Corp. (Houston, TX, USA). For liver triglyceride content measurement, 50 mg of frozen liver tissue per sample was weighed on dry ice, and its saponified extract was extracted using a published protocol [18]. Triglyceride levels were then quantified by Cobas c111 analyzer.

Caffeic acid, trigonelline hydrochloride, cafestol acetate, and ferulic acid were purchased from Sigma-Aldrich (St. Louis, MO, USA) and isotope-labeled hippuric acid (Ring-13C6, 99%) from ReseaLife Chem Science (Burgdorf, Switzerland). HPLC-grade acetonitrile, methanol and acetic acid were purchased from Fluka (Sigma-Aldrich, St. Louis, MO, USA).

### 2.7. Histopathological Studies

The right anterior lobe of the liver was harvested from live anesthetized animals and fixed immediately in 10% neutral buffered formalin. Tissues were then embedded in paraffin and cut into 4-mm slices using an automatic microtome. Sections were mounted on slides and stained with hematoxylin and eosin, and Masson-trichrome. Samples were scanned at low-power, and studied in-detail in five random medium-power fields. A representative field was randomly selected from each group to make the photographs. Steatosis was categorized as large-droplet macrovesicular, small-droplet macrovesicular, and microvesicular, and graded on the scale of 0–3 based on the percentage of hepatocytes containing fat vacuoles: grade 0 < 5%, grade 1 = 5–33%, grade 2 = 34–66%, and grade 3 > 67%. All histologic analyses were performed by an experienced histopathologist in a blinded manner. 

### 2.8. Hyperpolarized-MR Spectroscopy Examination

Rats with a weight range of 400 to 660 g were scanned in a 3T GE clinical system (GE Healthcare, Milwaukee, WI, USA) equipped with a dual tuned ^13^C/1H volume rat coil (GE healthcare, Brøndby, Denmark). Hyperpolarized [1-^13^C]pyruvate was prepared and polarized in a SpinLab system (GE Healthcare, Milwaukee, WI, USA) in accordance with the standard protocol [19]. In brief, rats were anesthetized with sevoflurane (3% sevoflurane in 2 L/min air), and a tail vein catheter (0.4 mm) was inserted for injection of [1-^13^C]pyruvate. Upon full polarization (>35%) 1.5 mL (37 °C, pH 7.4) isotonic [1-^13^C]pyruvate solution was injected over 15 s. Anatomical 1H imaging was used for positioning the ^13^C imaging plane. A T2-weighted fast spin echo sequence was used in the axial and coronal orientation covering the liver. Following the anatomical scout, an axial oblique slice-selective (10 mm, 10°) ^13^C-dynamic time series with a repetition time of 1 s (total 120 s, one image/s) was performed. The sequence was initiated before the injection of [1-^13^C]pyruvate. Each individual peak area was fitted using a general linear model fit on the time-domain data, followed by a model-free ratio-metric analysis of the AUC product and substrates.

### 2.9. Liquid Chromatography Tandem Mass Spectrometry (LC-MS/MS)

Caffeic, ferulic, and hippuric acid (internal standard, IS) were dissolved in absolute methanol as stock working solutions, and were kept at −80 °C. Calibration curves were prepared in the concentration range of 0.0122–6.25 ng/mL in 1% acetic acid solution. IS was added to the calibration curves to a final concentration of 5 ng/mL. The analyte/IS concentration ratio was plotted against the analyte/IS peak area ratio as a linear regression curve with 1/× weighting. Caffeic acid and ferulic acid were extracted from plasma samples using the liquid–liquid extraction method with methanol. We used 5 µL and 10 µL of plasma for the extraction of total and free forms of the compounds, respectively. Sample hydrolysis was performed with 20 µL of enzyme mix as described [20]. Protein precipitation and compound extraction were done using 200 µL of methanol. After centrifugation for 5 min at 20,800× *g*, the supernatant was transferred to a vial and dried under N_2_ flow at 30 °C. The precipitate was reconstituted in 500 µL H_2_O containing 1% acetic acid. Samples were measured on MicroLC 200 Series from Eksigent/AB Sciex (Redwood City, CA, USA) equipped with a Synergi Polar-RP Column, 500 mm × 1 mm with 4.0 µm particle size from Phenomenex and QTrap 5500 mass spectrometer from AB Sciex (Framingham, MA, USA). The chromatographic separation was performed according to the protocol by Nørskov et al. [20]. Solvent A was water and Solvent B was acetonitrile, both containing 20 mM of acetic acid. The column was equilibrated for 2 min at 10% of solvent B. Five µL of sample was injected by an autosampler and separation was achieved by using a step gradient starting at 10% B, held constant for 0.6 min, increased to 40% over 1.5 min, kept constant for 1 min, and then increased to 60% over 1.5 min, with a flow rate of 65 µL/min and a column temperature of 30 °C. The mass spectrometer was equipped with an ESI source with the following settings operated in negative ionization mode: curtain gas 20 psig, Gas 1 60 psig, Gas 2 60 psig, temperature 475 °C, and ionization spray operated at −4500 eV. Flow injection analysis was performed to optimize the turbo V source of the instrument. The ESI source deprotonated molecules were detected in multiple reaction monitoring (MRM) mode. The compound-dependent parameters were optimized for each compound by syringe infusion of pure standard and are listed in Table S1 (Supplementary Materials). Data analysis was performed in Analyst software 1.6.2 from AB Sciex (Framingham, MA, USA). Plasma from control rats was used for validation of the method. We pooled the plasma and aliquoted in several tubes which were kept at −80 °C as quality control samples (QC). Spiked QC samples at three concentrations, low (0.195 ng/mL), medium (1.56 ng/mL) and high (6.25 ng/mL), and five replicates were used to calculate the recovery of caffeic and ferulic acids (Table S2 in the Supplementary Materials). Using QC samples, batch-to-batch variation (*n* = 5) was calculated to be less than 10%. The matrix effects were validated in both pure solvent and QC samples and showed to have no discernible effects.

### 2.10. Statistical Methods

Data are presented as means ± standard error of the mean (SEM) or standard deviation (SD) unless stated otherwise. Normality of data was checked using quantile–quantile (q–q) plots and Shapiro–Wilk normality test, and variance homogeneity was tested by the Levene’s test. Independent samples Student’s *t*-test was used to test the significance of differences between the two groups. The Cohen’s *d* was calculated to estimate the effect size of significant findings. All the analyses were performed at a two-sided significance level of 0.05 using IBM SPSS Statistics for Windows (Version 22.0. IBM Corp., Armonk, NY, USA). Area under the curve (AUC) calculations were executed using GraphPad Prism for Windows (version 5.01, GraphPad Software, La Jolla, CA, USA).

## 3. Results

### 3.1. The Chronic Study

#### 3.1.1. Energy Homeostasis

**Figure 1:**
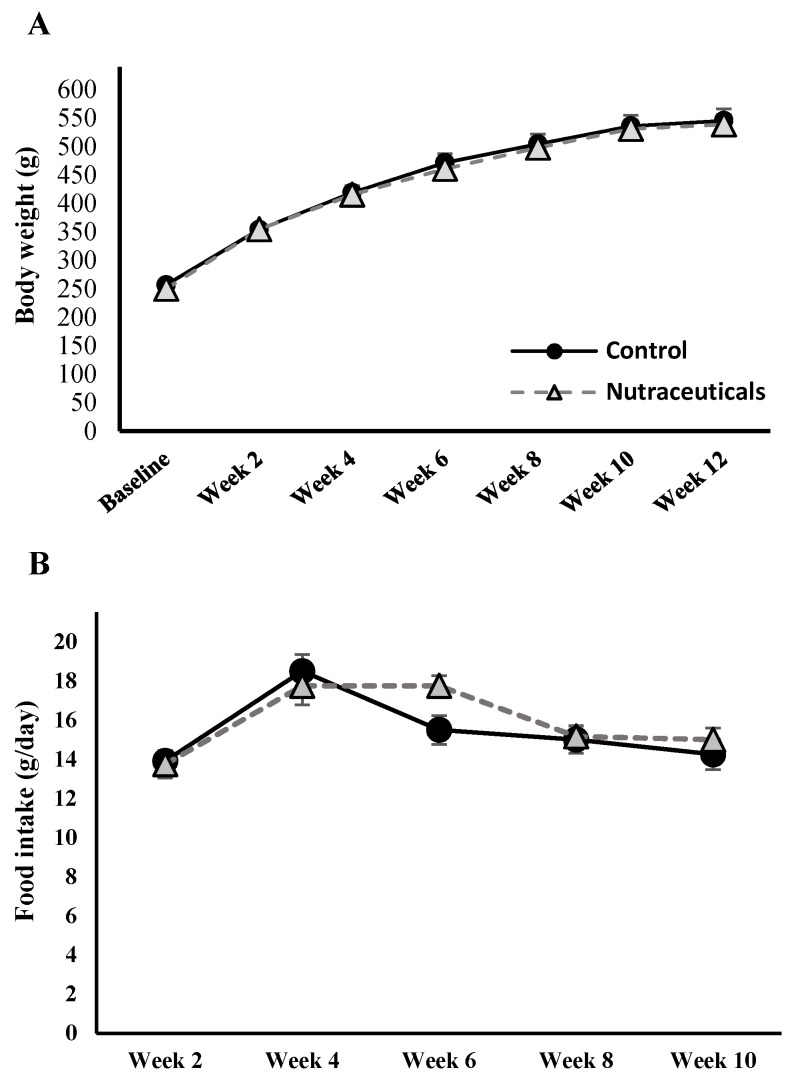
(**A**) Body weight of high-fat/high-fructose-fed rats in both study groups; (**B**) food intake was measured in one day every second week from week 2 to 10, and presented as g/day. No significant difference was found between groups (error bars: standard error of the mean). Nutraceuticals combination consisted of caffeic acid, trigonelline, and cafestol.

As seen in Figure 1, rats in both groups consumed similar amounts of food per day and gained weight at comparable rates over the intervention period. 

#### 3.1.2. Blood Glucose and IR

**Figure 2:**
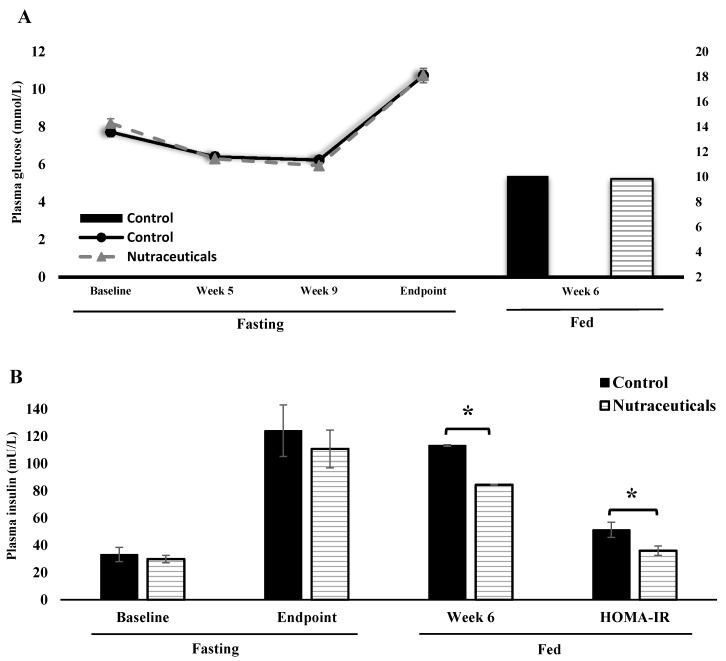

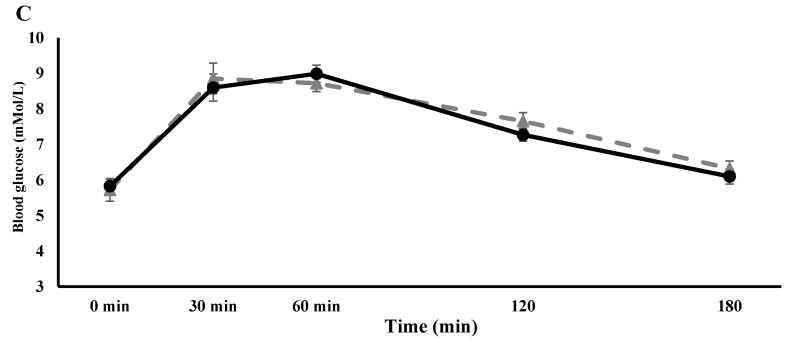
Insulin and glucose homeostasis indices from the chronic study. (**A**) The line graph presents mean fasting plasma glucose and the bar graph shows fed plasma glucose measured at mid-study; (**B**) Fasting and fed plasma insulin levels. HOMA-IR in fed state was calculated from fed glucose and insulin values. Asterisk indicates a significant difference between groups (*t*-test, *p* < 0.05); (**C**) Blood glucose measured by glucometer after a glucose load during an OGTT.

Blood glucose was measured four times in the fasting, and once in the fed state at mid-study (Figure 2). Rats in the two groups had similar blood glucose levels in fasting and fed states at all time points. Fasting blood glucose rose steeply from week 9 to 12 (Figure 2A). The average level of glycemia as measured by HbA_1C_ was unaffected by the nutraceuticals mixture compared to the control diet (Table 1). After 12 weeks on HFHF diet, the mean fasting plasma insulin levels increased dramatically to 3.7 times the baseline levels in both groups. Although fasting levels were not different between groups, fed insulinemia was reduced significantly in the intervention group (mean difference = 30.80, 95% CI = 0.95–60.64) at mid-study (*t* = 2.152, *p* = 0.044, Cohen’s *d* = 0.96), which led to lower HOMA-IR index in the fed state (*t* = 2.283, *p* = 0.033, Cohen’s *d* = 1.00) (Figure 2B). As shown in Figure 2C, an oral glucose load caused similar plasma glucose peaks in both groups.

#### 3.1.3. Plasma Lipids and Cytokines

Data for the main classes of plasma lipids measured at baseline and endpoint are presented in Table 1. There were no notable differences between groups except for a dramatic drop in the plasma level of NEFAs in both groups compared to the baseline (*p* < 0.0001 in both groups). HDL-C at endpoint tended to be higher in the nutraceuticals group, but this was not statistically significant. The average adiponectin level was 18% higher in the nutraceuticals group after 12 weeks of intervention (*t* = −2.097, *p* = 0.048, Cohen’s *d* = 0.92). IL1b plasma levels in the nutraceuticals group at endpoint was not significantly lower (Table 1). 

#### 3.1.4. Liver Steatosis

**Figure 3:**
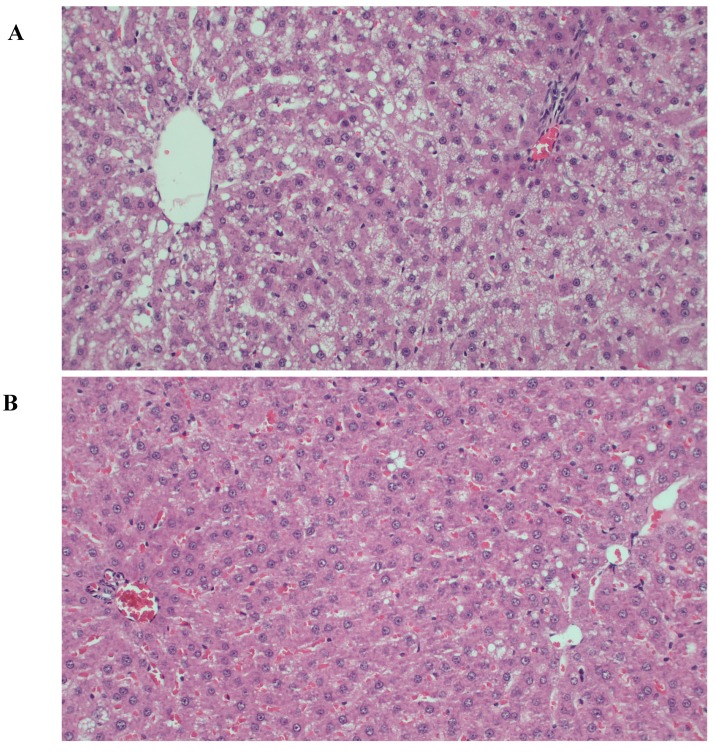
Representative histological liver sections (magnification 20×): (**A**) A sample from the control group with marked fatty change showing mixed small- and large-droplet macrovesicular steatosis; (**B**) A sample from the nutraceuticals group showing mild large-droplet macrovesicular steatosis.

As displayed in Figure 3, there were mixed small- and large-droplet macrovesicular steatotic changes in hepatocytes of the two groups. Semiquantitative scoring of steatosis yielded a mean score of 1.42 (SD = 1.31) in the control, and 1.18 (SD = 0.98) in the nutraceuticals group. 58% and 36% of samples from the control and nutraceuticals groups, respectively, demonstrated severe steatosis (grade 2 or higher). Quantitative measurement of liver triglyceride content also confirmed the tendency of lower steatosis in the nutraceuticals group. Quantification of the ALT in plasma showed significantly lower levels in the nutraceuticals group at endpoint (*t* = 2.690, *p* = 0.014, Cohen’s *d* = 1.20) (Table 1). There was limited inflammatory cells infiltration throughout the lobules in both groups and no signs of fibrosis. 

#### 3.1.5. Hyperpolarized-[1-^13^C]Pyruvate MR Spectroscopy

**Figure 4:**
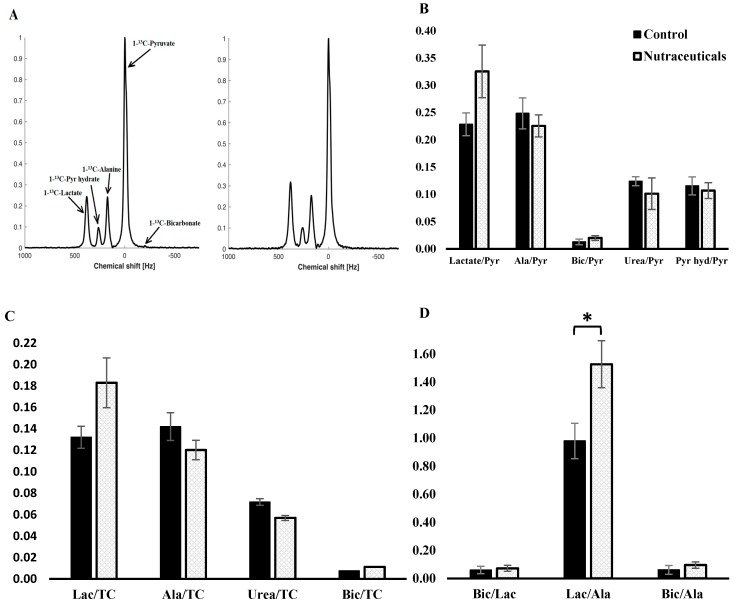
Liver ^13^C-MR spectroscopy: (**A**) Average sum spectrum of each group reconstructed from the dynamic spectra covering 120 s time span after the intravenous injection of hyperpolarized [1-^13^C]pyruvate. Peaks from left to right are [1-^13^C]-lactate, -pyruvate hydrate, -alanine, -pyruvate and –bicarbonate; (**B**) The area under the curve of signal intensity time curves for each metabolite normalized to [1-^13^C]pyruvate and (**C**) total carbon; (**D**) Comparison of liver signal intensity ratios of [1-^13^C]pyruvate metabolites between groups. Asterisk denotes a significant difference compared to the control group (*t*-test, *p* < 0.05).

Subsequent to the rapid injection of hyperpolarized-[1-^13^C]pyruvate, serial MR spectra were acquired every second for one minute. The summed spectra from each group are presented in Figure 4A. As displayed, signals from Hyperpolarized-[1-^13^C]pyruvate, -pyruvate hydrate, -lactate, -alanine, -bicarbonate, and -urea (thermal phantom) were identified in the spectrum. For statistical comparison between groups, the AUC of signal intensity time curves of each metabolite was calculated and normalized to pyruvate AUC and total carbon pool (Figure 4B,C, respectively). What stands out is the tendency towards increased lactate and decreased alanine ratios to pyruvate and total carbon in the nutraceuticals group. This gives rise to a significant shift in lactate/alanine ratio in the nutraceuticals group compared to the control group.

#### 3.1.6. Plasma Levels of Caffeic acid Metabolites

**Figure 5:**
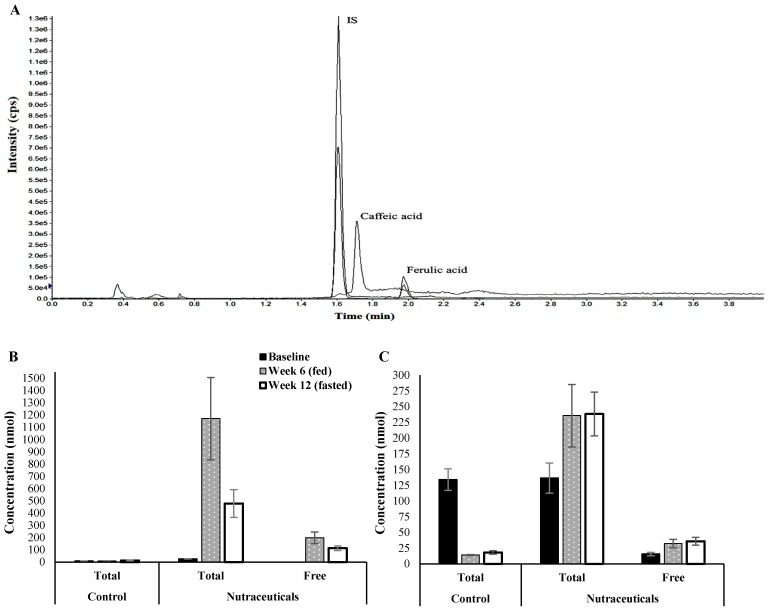
Quantitative HPLC-MS/MS data: (**A**) extracted ion chromatograms of caffeic acid, ferulic acid and internal standard in plasma; (**B**) plasma levels of total and free caffeic acid and (**C**) ferulic acid at different time points in both study groups calculated by an external standard calibration method. Error bars represent ± standard error of the mean.

Quantification was performed by LC-MS/MS to evaluate the efficiency of caffeic acid delivery in drinking water. Figure 5A presents an example of the chromatographic profile of a plasma sample. The same Figures also depicts the concentrations of total and free caffeic and ferulic acids in plasma samples taken at random time points in fed and fasting states. As shown, caffeic acid was almost absent in samples from the control group. Conjugated metabolites were the dominant forms of caffeic acid in both fed (~83% of total) and fasted (~76% of total) states. Although both total and free forms tended to be higher in the fed state, the differences were not statistically significant. The relatively high ferulic acid levels in both groups at baseline declined in the control group in later measurements due to shifting to a diet deficient in cereals. In contrast, ferulic acid levels increased in the plasma of the intervention group (Figure 5C). According to our data, less than 16% of the total ferulic acid on average was unconjugated in the peripheral circulation. This ratio was not affected by the feeding state of the animals.

### 3.2. Acute Study

**Figure 6:**
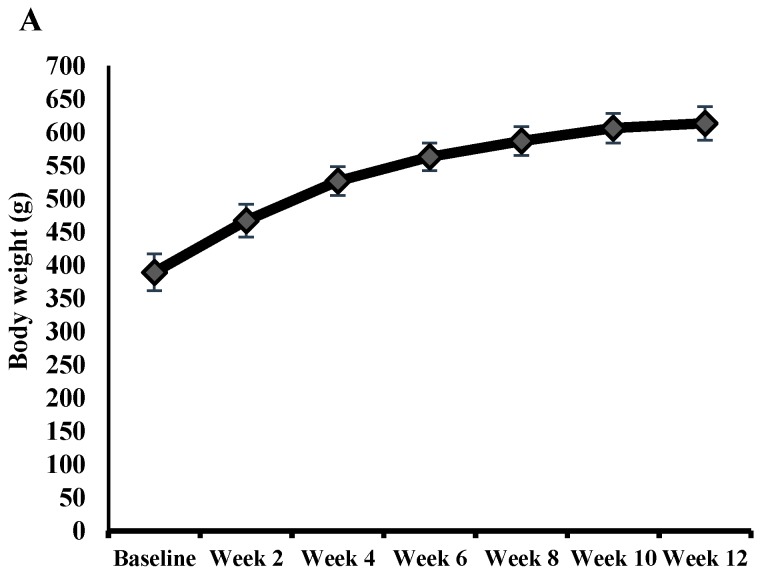

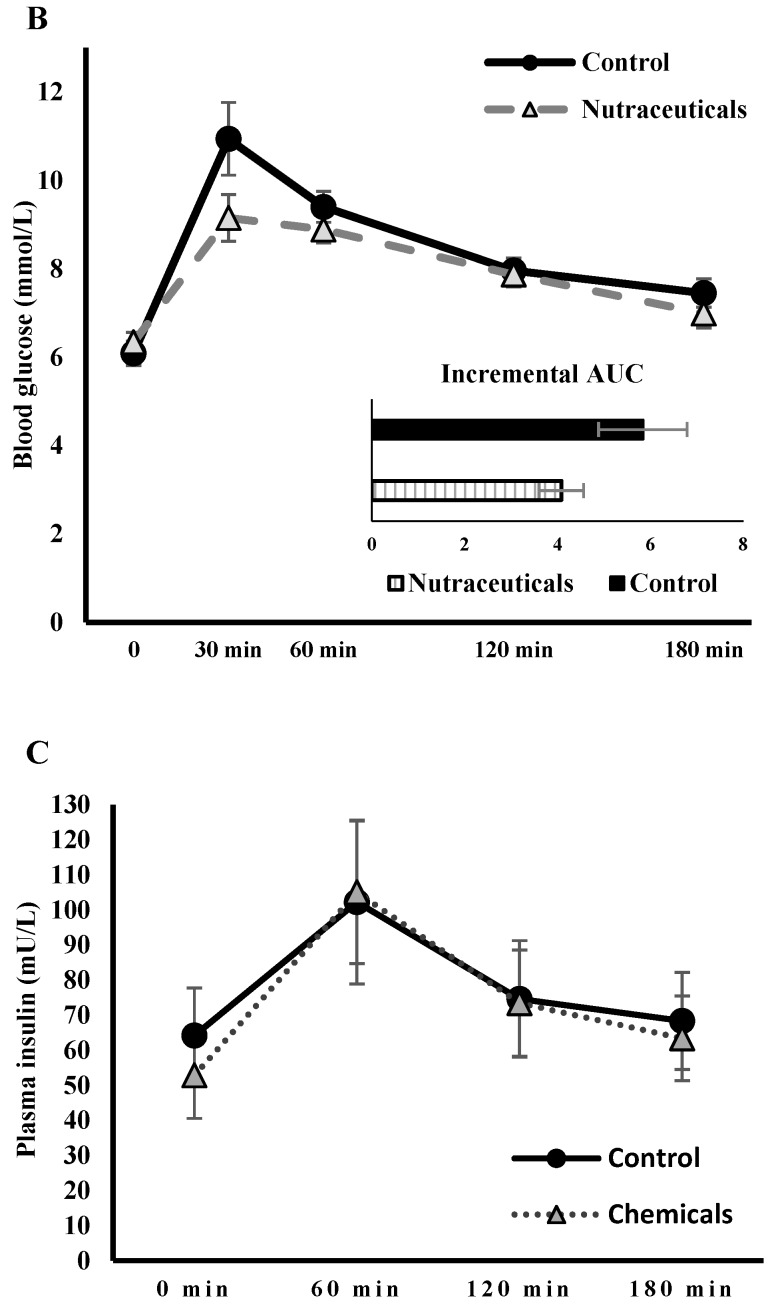
Results from the acute cross-over study. (**A**) Growth chart of the 11 rats used in the acute study; (**B**) Mean blood glucose levels during the OGTT (started 90 min after receiving a placebo or pellets supplemented with coffee chemicals) are plotted against time on the upper part. Under the line graph is a bar chart that displays its incremental area under the curve in each group; (**C**) The insulin response measured in plasma every hour after the glucose challenge.

Rats used in this part of the study had higher baseline and endpoint body weight compared to the chronic experiment due to their higher age (Figure 6A). In a glucose challenge test, blood glucose peak and incremental AUC of glucose response tended to be lowered by nutraceuticals; however, this was not statistically significant (*t* = 1.645, *p* = 0.117) (Figure 6B). In contrast, plasma insulin levels measured hourly after the glucose load were not altered by the intervention (Figure 6C).

## 4. Discussion

We studied the effects of a combination of coffee nutraceuticals on the key parameters of diet-induced MetS in rats using a range of standard and state-of-the-art assessment methods. HFHF-fed rats appropriately reflect the Western diet-induced MetS and NAFLD. Since a similar chemical combination has never been used in an in vivo setting, we compared our findings to the previous studies that used decaffeinated coffee and a comparable animal model (Table 2). 

Based on evidence from population-based studies showing a significant inverse association between moderate to high (≥4 cups/day) coffee consumption and future risk of the MetS [8], we administered the nutraceuticals in a dosage corresponding to high–moderate coffee consumption (6–8 cups/day) in an adult human. Half of this amount was used as a single-dose in the acute study. A continuous delivery method via drinking water was favored over single daily dosing due to a relatively short plasma half-life of caffeic acid and trigonelline. The plasma levels of metabolites achieved by continuous administration of 30 mg of caffeic acid in 24 h, compared with the levels reported previously after giving a 25-mg single dose [27], indicate that a reasonable delivery of compounds is achieved via drinking water in the present study.

The observation in our study that coffee nutraceuticals did not alter weight gain rate nor food intake support the findings of Song et al. [10]. The authors have proposed the minimum effective dosage of decaffeinated coffee bean extract (DCBE) on energy homeostasis to be 0.3% *w*/*w* of food, approximately equal to 9 mg per day in a mouse, which is higher than the dosage we used in the present study (Table 2). Pure 5-CQA, the most abundant CQA isomer in coffee, however, was effective in comparable dosage to caffeic acid in our formulation, which points to a higher efficacy [10]. Studies which used lower concentrations [22,24] or shorter intervention periods [26] did not detect any significant effect on weight development. Two obesity-related cytokines were measured at endpoint: adiponectin and IL_1b_. Adiponectin is an adipokine released from the adipose tissues in response to caloric restriction. There is evidence suggesting that adiponectin is positively associated with insulin sensitivity, and negatively correlated with body weight and fat accumulation [28]. Higher secretion or reduced clearance of adiponectin after long-term consumption of coffee nutraceuticals indicates that these chemicals may favorably influence adipose tissue metabolism, and may modify the development of some obesity complications e.g., IR. A similar positive association between coffee consumption and plasma adiponectin was also reported in humans [29]. IL_1b_, a pro-inflammatory cytokine, was quantified to evaluate the potential anti-inflammatory properties of coffee compounds. However, in the present study, no significant effect was detected.

The considerable increase in fasting blood glucose in both groups after week 9 was plausibly driven by an increased IR and glucose intolerance induced by the HFHF diet, as shown previously [16]. Lower levels of insulin were required to control blood glucose in the fed state in the intervention group compared to the control group. This points indirectly to higher insulin sensitivity. This was supported by the HOMA-IR calculations. Supposedly, the effect size of our nutraceuticals mixture was not big enough to be reflected in other insulin sensitivity tests such as the OGTT and fasting insulinemia. This is comparable to what other authors reported with lower or comparable intervention dosages [10,22,23]. In the case of uncontrolled and prolonged oxidative stress disrupting insulin function [30], insulin sensitizing efficacy can be increased by the potent antioxidant activity of the coffee nutraceuticals, especially caffeic acid [31], along with the effect of cafestol on glucose uptake [14]. The role of treatment duration on glucose homeostasis can be inferred from the significant improvement of glucose tolerance with long-term administration of a relatively low dose of DCBE in a study by Ho et al. [24]. Insignificant changes are also observed with short-term treatment with high doses [25]. After acute oral administration of coffee nutraceuticals, we observed an insignificant decline in glucose and no effect on insulin response. It was reported previously that 36 mg of 5-CQA given by gavage together with a mixed meal decreased glucose but not insulin response in non-obese rats [32]. Pretreatment with intravenous caffeic acid (1 mg/kg) diminished glycemia after a parenteral load of glucose in healthy rats [33]. The relatively low oral bioavailability of caffeic acid may explain the inferior effectiveness of oral versus intravenous administration.

Our mixture of nutraceuticals failed to shift the rats’ plasma lipid profile in contrast with what has been reported with chlorogenic acids and DCBE [10,34]. This was presumably due to the resistance of rats to the cholesterol-raising effects of cafestol [35], low dosage [10], and the lack of compounds such as melanoidins, which showed effectiveness in reducing plasma triglycerides [36]. The considerable reduction in circulating NEFAs may have been caused by the substantial hyperinsulinemia in both groups in the fasting state, which can suppress the release of NEFAs from adipose tissues.

Effects of coffee nutraceuticals on NAFLD as a feature of the MetS is of special interest. The composition and dosage we used in this study were enough to visibly diminish the grade of fatty change in the liver, and tended to reduce the fat content of hepatocytes. In previous in vivo studies, higher dosages of DCBE and coffee polyphenols showed significant potency in decreasing lipid content and steatotic changes of hepatocytes in HF-fed rodents [34,36]. Data from interventional clinical research on the effects of coffee compounds on NAFLD patients is not available at this time. We also utilized Hyperpolarized-[1-^13^C]pyruvate MR spectroscopy in order to assess the effects of coffee compounds on intrahepatocellular carbohydrate metabolism. Hyperpolarization of ^13^C through the process of dynamic nuclear polarization increases the signal-to-noise ratio of MR spectroscopy by more than 10,000 times in the liquid-state, and enables metabolic imaging of intermediary metabolites in the living subjects [37]. In our study, we spotted that the share of alanine in the pool of pyruvate metabolites in the nutraceuticals group is reduced. This change can be translated into a modified activity of ALT in the liver, which is compatible with the diminished ALT activity in the intervention rats’ plasma. Since the in vivo level of hepatic alanine increases with the progression of diet-induced liver steatosis [38], this finding may be a signal of a hepatoprotective mechanism of the nutraceuticals. Whether the rising trend in lactate/pyruvate ratio in the intervention group resulted from an inhibitory effect on downstream gluconeogenesis similar to that reported with metformin [39], or is caused by an increased lactate dehydrogenase flux secondary to ALT inhibition, is yet to be elucidated. There were no significant differences in bicarbonate conversion rate, an indicator of pyruvate dehydrogenase complex activity, between groups. Knowing that fasting can influence the rate of liver pyruvate metabolism [40], we examined all animals in the fed state.

Critical perspectives: (1) Viewing the study retrospectively, it may be a point of contention whether replacing all carbohydrates in a HF diet with fructose makes a more effective HFHF diet compared to the standard form of HFHF; (2) For future research, mixing nutraceuticals with food appears to be a superior option in favor of both dosing accuracy and avoiding uncertainty with solubility and precipitation; (3) Caffeine is a significant part of the coffee chemical composition, and long-term caffeinated coffee has exhibited a multiplicity of benefits on different aspects of human health despite its aggravating short-term effects on insulin sensitivity [41]. A similar caffeinated formula might have higher long-term efficacy in improving the metabolic state of MetS subjects; (4) Higher effect size and broader impact of CQAs on IR and lipid metabolism [10,21] compared to what we observed with similar doses of caffeic acid raise a question regarding the generally-accepted notion of 2:1 metabolic equivalency of CQA to caffeic acid. This warrants further investigation; (5) The present study did not explore the molecular action of the coffee compounds. Further molecular assessments need to be considered in future studies.

## 5. Conclusions

Long-term administration of a combination of nutraceuticals from the three main groups of coffee compounds provided an improvement in insulin sensitivity in the fed state, and increased the plasma levels of adiponectin. Decreasing hepatocyte damage correlated with a decline in intrahepatocellular and circulating ALT activity. This points to a level of hepatoprotection against steatosis, with modest histological changes. We presume that the daily dose of nutraceuticals utilized in this study touches upon their minimum effective dosage. Higher dosages and/or longer periods of administration are likely to impact broader aspects of the diet-induced MetS with improved efficacy.

## Figures and Tables

**Table 1:** Results of plasma biochemistry tests performed before and after the intervention presented as mean (SEM). Significant *p*-values in the between-group analysis are in bold.

	Control Group		Nutraceuticals Group		*p*-Value of Change/Difference
	Baseline	12 Weeks	Baseline	12 Weeks	
**Plasma lipids (mmol/L)**					
**Total cholesterol**	1.87 (0.08)	2.00 (0.08)	2.02 (0.10)	2.33 (0.16)	0.428
**HDL-C**	1.42 (0.05)	1.58 (0.06)	1.55 (0.07)	1.83 (0.11)	0.492
**LDL-C**	0.48 (0.03)	0.36 (0.03)	0.47 (0.03)	0.49 (0.07)	0.097
**Triglyceride**	0.36 (0.02)	0.42 (0.05)	0.37 (0.01)	0.39 (0.04)	0.675
**NEFAs**	1.01 (0.08)	0.31 (0.03)	0.91 (0.05)	0.31 (0.02)	0.268
**HbA_1C_ (mmol/mol)**	-	23.08 (0.29)	-	23.55 (0.55)	0.451
**Plasma adiponectin (µg/mL)**	-	4.40 (0.28)	-	5.39 (0.39) *	**0.048**
**Plasma IL_1b_ (pg/mL)**	-	13.65 (1.88)	-	8.92 (2.46)	0.139
**Plasma ALT (U/L)**	31.20 (0.73)	27.72 (1.39)	32.78 (0.71)	22.97 (1.09) *	**0.004**
**Liver triglycerides content (mmol/L)**	-	2.00 (0.26)	-	1.62 (0.17)	0.223

* Significantly different from the control group (*p* < 0.05). Abbreviations: HDL-C: high-density lipoprotein cholesterol, LDL-C: low-density lipoprotein cholesterol, NEFAs: non-esterified fatty acids, HbA_1C_: Glycated hemoglobin, IL_1b_: interleukin-1, ALT: alanine transaminase

**Table 2:** A comparison of intervention dosage and duration of the present study and selected published studies on hydroxycinnamic acids and/or decaffeinated coffee extract. Human equivalent dosage enables the comparison between different species used in different studies. For the present study, the dosage of both caffeic acid and total compounds are presented to facilitate the comparison.

	Model Species	Intervention Duration (Weeks)	Intervention	Dosage (mg/Day)	Human Equivalent Dosage
Present study	Sprague-Dawley rat	12	Chemicals combination	51	1428
			CA	30	840
Song et al., 2014 [10]	C57BL/6N mouse	11	DCBE	3	507
				9	1521
				27	4563
			CGA	4.5	761
Peng et al., 2015 [21]	Sprague-Dawley rat	12	CGA	7	196
				32	896
Mubarak et al., 2013 [22]	C57BL6 mouse	12	CGA	0.3	51
Li Kwok Cheong et al., 2014 [23]	C57BL6 mouse	12	DCBE	15	2535
Ho et al., 2012 [24]	C57B6SJL mouse	20	DCBE	2.8	473
Jia et al., 2014 [25]	C57BL/6J mouse	9	DCBE	60	10,140
Shearer et al., 2007 [26]	Sprague-Dawley rat	4	DCBE	1600	44,800

Assumptions: human/mouse conversion factor = 169; human/rat conversion factor = 28; high-fat-fed mouse body weight = 35 g; mouse food intake = 3 g/day; Acronyms: CA: caffeic acid; DCBE: decaffeinated coffee bean extract; CGA: chlorogenic acids.

## Supplementary Materials

The following are available online at www.mdpi.com/2072-6643/10/1/6/s1, Table S1: Compound-dependent LC-MS/MS parameters, declustering potential (DP), entrance potential (EP), collision energy (CE) and cell exit potential (CEP), Table S2: Recovery percentile of caffeic and ferulic acid after sample pretreatment at three spiked concentrations: low (0.195 ng/mL), medium (1.56 ng/mL) and high (6.25 ng/mL) (*n* = 5).
