# Supplementary material for: A Combination of Coffee Compounds Shows Insulin-Sensitizing and Hepatoprotective Effects in a Rat Model of Diet-Induced Metabolic Syndrome
Source: Nutrients. 2017 Dec 22;10(1):6. doi: 10.3390/nu10010006 (PMC5793234; doi:10.3390/nu10010006)
Supplement: Supplementary file 1 [file nutrients-10-00006-s001.pdf]

Supplementary Materials:

Table S1. Compound-dependent LC-MS/MS parameters, declustering potential (DP), entrance potential (EP), collision energy (CE) and cell exit potential (CEP).

	**Q1 mass**	**Q3 mass**	**DP**	**EP**	**CE**	**CEP**
Caffeic acid	179.0	135.0	-35	-10	-21	-11
Ferulic acid quantifier	193.0	134.0	-52	-10	-21	-9
Ferulic acid qualifier	193.0	178.0	-52	-10	-17	-15
IS quantifier	184.0	139.8	-43	-10	-16	-10
IS qualifier	184.0	82.8	-43	-10	-22	-9

Table S2. Recovery percentile of caffeic and ferulic acid after sample pretreatment at three spiked concentrations: low (0.195 ng/mL), medium (1.56 ng/mL) and high (6.25 ng/mL) (n = 5).

	**Recovery %**		
	**Low**	**Medium**	**High**
**Caffeic acid**	69.2 ± 3.6	82.9 ± 2.1	74.6 ± 1.2
**Ferulic acid**	100.7 ± 1.9	92.4 ± 1.7	88.4 ± 1.0
